# Promoting Protein Intake in an Ageing Population: Product Design Implications for Protein Fortification

**DOI:** 10.3390/nu14235083

**Published:** 2022-11-29

**Authors:** Victoria Norton, Stella Lignou, Lisa Methven

**Affiliations:** Department of Food and Nutritional Sciences, Harry Nursten Building, University of Reading, Reading RG6 6DZ, UK

**Keywords:** protein, fortification, older adults, product design

## Abstract

Protein is a macronutrient of interest for an ageing population and intake requirements increase with age. Accordingly, protein is often fortified into products for older adults to help alleviate malnutrition and impede sarcopenia. However, more emphasis needs to be placed upon designing protein-fortified products to ensure suitability for older adults. This study involved a two-stage approach: (1) an initial review of products commonly fortified with protein and (2) two questionnaires for younger and older adults (*n* = 73; 18–30; 65+) to investigate optimal portion sizes (drinks and cakes) as well as attitudes, consumption habits and preferences towards protein fortification. The initial literature and market review demonstrated protein-fortified products are typically in liquid or snack format; however, there is considerable variability in terms of product types, serving size and protein sources. There were no age-related differences found for ideal cakes portion size whereas there were for liquids. Older adults are typically not consuming protein-fortified products; therefore, more importance should be placed on the consumption moment (breakfast or as snacks between meals) and on cereals, pasta, porridge, cakes, and biscuits. Older adults need increased awareness of, and more education on, the benefits of protein consumption, coupled with products tailored and designed to encourage intake.

## 1. Introduction

It is recognised that we have an ageing population in the UK resulting from improvements in healthcare, lifestyle and technology compared with previous generations [1]. However, the ageing process can be influenced by physical, social, and psychological factors, all of which can lead to an increased risk of poor nutritional status [2]. This supports the importance of ensuring products are developed to be suitable for older adults to encourage food intake, especially as nutritional provision can enhance functional and clinical outcomes [3]. Protein is of particular interest for an ageing population; intake requirements are considered to increase with age (1.0–1.2 g/kg/day) due to anabolic resistance (blunted muscle protein synthesis response) and increased metabolism resulting from inflammatory conditions [4,5]. Therefore, sufficient protein intake can help prevent age-related muscle mass, strength, and functional losses [5]. However, older adults can often struggle to consume adequate quantities of protein and this prevalence is relatively widespread given that 65–67% of community based older adults are below the suggested 1.2 g/kg/day threshold [4,5,6]. This can be due to a variety of reasons including: small appetites, physical challenges (impacting food preparation/consumption which can alter food choices), financial and social constraints and protein intake typically being centred around one meal [4,5]. Accordingly, protein is often fortified into products for older adults to help alleviate malnutrition and impede sarcopenia.

Dietary proteins are frequently cited for their functionality benefits in developing products, such as heat stability, foaming, water binding, solubility, gelation, and emulsification [7]. However, when designing products for older adults the suitability of the protein source in terms of nutritional, functionality and sensory aspects must also be considered to maximise uptake. Animal derived proteins (such as whey proteins) have well-cited nutritional, functional and health benefits; accordingly, they are regularly fortified into products and/or used to formulate oral nutritional supplements (ONS) for older adults [8,9,10,11,12,13]. Additionally, whey protein may be less satiating in older adults compared with younger adults; therefore, enabling increased protein intake without potentially impacting subsequent food intake [14,15,16]. However, whey proteins can be associated with negative sensorial issues (flavour and texture) as highlighted in recent reviews [17,18,19].

Product compliance is fundamental to improving protein intake in an ageing population. Hubbard et al. demonstrated compliance ranging from 37% to 100% in a systematic review of ONS; therefore, highlighting variability and challenges [20]. Moreover, high wastage can result in cost implications [21]; accordingly, ensuring older adults are presented with products which encourage intake is key. Design pointers that can improve food intake include: energy/nutrient dense fortification, suitable portion sizes, diet/product variability and palatable/appetising products [22]. Additionally, fortifying familiar and popular foods with protein could be a viable solution to increasing intake and promoting liking in an ageing population [23,24,25,26,27,28,29]. Moreover, older adults often have various age-related changes which subsequently impact product compliance, sensory perception, and food intake. For example, older adults often have reduced appetite, modulated sensory sensitivity and oral impairments; all factors which can influence the eating experience and/or protein intake [29,30,31,32,33]. Such factors need to be considered and the protein content and portion size subsequently optimised in order to maximise product consumption as they present key challenges in designing protein-fortified products for older adults.

The main nutritional approaches to enhance protein intake within the ageing population are supplementation and fortification. Typically, either ONS (providing additional micro-and macro-nutrients) or fortified snacks (adding protein content without increasing portion size) are utilised between and/or after meals [34]. More generally, a product needs at least 12% or 20% energy content from protein to be considered as a protein source or high protein source, respectively [35]. Within clinical settings protein content in products is often variable (depending on the exact purpose); however, ONS usually have at least 5.0 g of protein per 100 g [36]. Similarly, the British Dietetic Association (BDA) suggests between meal snacks (at least two daily) in hospitals should have 2.0 g and 4.0 g protein (per portion) for nutritionally well and vulnerable individuals, respectively [37].

A food first approach (such as small/regular meals with nutrient dense foods, nutritious liquids, high energy/protein foods into the diet and/or as snacks between meals) is a key strategy to promote food intake [37]. Protein-fortified products fit this remit well, but such products encompass a broad range (e.g., snacks, main meals, desserts, finger foods, ONS, etc.) and are often targeted at varying markets (such as sport, health, lifestyle and/or older consumers) [38,39]. It is apparent that protein needs increase with age [4,5]; however, more emphasis needs to be placed upon designing products to meet older adults’ requirements. Studies have typically focused on understanding age-related differences or clinical outcomes rather than focusing on the product design perspective; accordingly, this warrants additional investigation. This paper aims to (1) explore types of food matrices commonly fortified with protein with an initial literature and market review; (2) determine consumers’ perceived ideal portion sizes for drinks and cakes and whether this varies between age groups; (3) understand attitudes, consumption habits and preferences towards protein-fortified products in both younger and older adults; and (4) provide practical solutions for future protein fortification of products.

## 2. Materials and Methods

### 2.1. Study Overview

**Figure 1:**
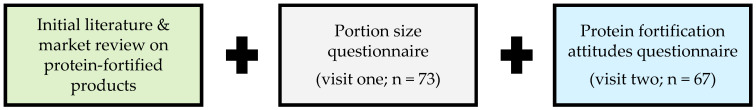
Study overview.

This study involved an initial literature and market review coupled with two questionnaires, as outlined in Figure 1. Seventy-three healthy (minimal medication—average number of medications: 1.68 ± 0.95 and community living) male (37%) and female (63%) consumers from two age groups (41 younger adults: 18–30 years, 25.7 ± 3.2 years and 32 older adults: over 65 years, 74.6 ± 5.7 years) completed a series of questionnaires as part of a single blinded crossover trial (University of Reading Ethics Committee—study number: UREC 19/67 and registered as NCT04302779 on www.clinicaltrials.gov) involving two study visits. It should be noted this study was carried out in February and March 2020; however, it stopped earlier than planned due to the impending COVID-19 pandemic, but sufficient power was obtained. Additional detail on the inclusion/exclusion criteria, study design and subsequent data relating to consumers’ liking and perception of protein-fortified cupcakes has already been published by Norton et al. [40]. The study had appropriate power to find differences in both product (protein fortification) and age (younger versus older adults) [40].

### 2.2. Initial Literature and Market Review on Protein-Fortified Products

This task aimed to explore different food matrices regularly fortified with protein in three key areas. (1) Literature-based studies—focusing on protein-fortified products in a range of settings. Papers written in English were reviewed from 2010 to 2020, searching for studies that had tested different types of protein-fortified products with older adults in any setting. Data was included from 17 papers published from 2014 to 2019. (2) Clinical settings—involving ONS products typically prescribed in clinical settings in the UK; and (3) retail market—covering protein-fortified products commonly purchased in the UK via supermarkets or popular on-line stores. This was to gain an initial understanding of products available to older adults and relevant background knowledge to design appropriate questionnaires for consumers. Data extraction focused on identifying examples of common products fortified with protein and corresponding protein sources, serving sizes and protein content (per serving size and per 100 g or 100 mL). The purpose of this task was not to complete an exhaustive review of protein products available but rather to understand key trends and identify challenges and pointers for future protein fortification of products.

### 2.3. Questionnaire Design

**Figure 2:**
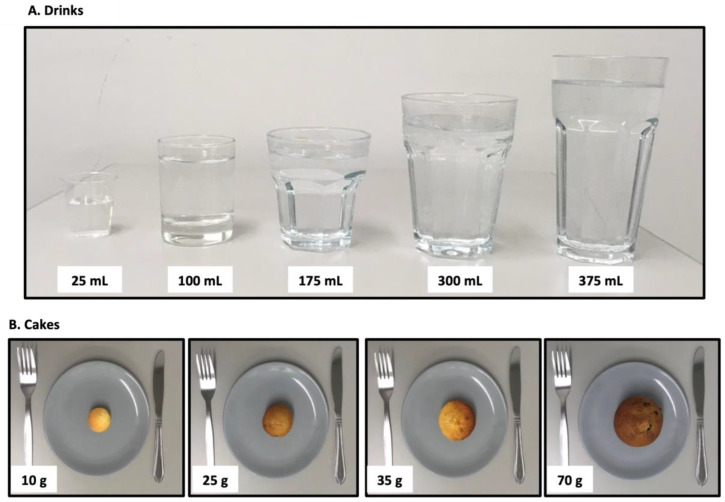
Drink and cake examples from the portion size questionnaire. Consumers circled what they considered to be an ideal portion size from a series of images and if the ideal portion size was not present they could add a comment.

Consumers independently completed two questionnaires, via paper format (to ensure suitability for older adults), during two separate visits. The initial literature and market review on protein-fortified matrices provided fundamental bases for the subsequent questionnaire development. The portion size questionnaire was designed to explore consumers’ ideal portion sizes relating to a series of popular and commonly consumed drinks and cakes in the UK. The rationale for selecting drinks and cakes was to reflect the common nutritional strategies used within an ageing population to increase protein intake: (i) drinks are typically given to older adults in the form of ONS to enhance nutritional status and (ii) cakes are well liked by older adults, can be easily consumed between meals, and can be readily fortified with protein to increase protein intake. Moreover, liquids and snacks represent typical product types used for older adults at risk of malnutrition, as identified in Section 2.2. Consumers were provided with images relating to seven drinks and five cakes with a corresponding portion size and were asked to circle their ideal portion size (Figure 2 and Table 1).

The protein fortification attitudes questionnaire aimed to understand consumption habits and preferences. The questionnaire focused on determining: (1) frequency of protein-fortified product consumption (single selection: never, monthly, one-to-three times a month, weekly, two-to-six times a week and more than once a day); (2) attributed importance for selecting protein-fortified products (ranking: appearance, smell, taste, flavour, texture and cost); and (3) preferences towards potential products for protein fortification (check-all-that-apply (CATA) where consumers ranked their four most preferred protein-fortified products from: biscuits, cakes, ice cream, chocolate, cereals, jelly, soups, angel delight, flapjack, pasta, sauces, savoury dips, rice pudding, pancakes, brownies and porridge). Consumers that regularly consumed protein-fortified products (e.g., monthly, one-to-three times a month, weekly, two-to-six times a week and more than once a day) were asked a series of follow up questions relating to current habits and experiences. For example, (a) typical timing of protein-fortified product consumption (CATA: breakfast, lunch, evening meal and snack); (b) examples of commonly consumed protein-fortified products (CATA: protein bars, yoghurts, cakes, biscuits, ice cream, soups, cereals, protein beverages and other); and (c) rating of protein-fortified products recalled taste/flavour and texture (single selection: very poor, poor, neutral, good and very good).

### 2.4. Statistical Analysis

Questionnaire data was considered non-parametric (based on normality of residues). The following statistical approaches were utilised in XLSTAT (version 2022.3.2.1348, New York, NY, USA) where a: (1) Mann-Whitney test was used to determine age-related differences relating to the drink and cake portion size data; (2) Kruskal-Wallis test was applied to overall drink and cake types, protein-fortified product preferences and commonly consumed protein-fortified products; (3) Chi-square test on contingency tables was deployed on category data (yes: I consume protein-fortified products and no: I do not consume protein-fortified products) to establish age-related differences; and (4) Friedman’s test was performed on ranked data and subsequent post hoc analysis (on significant results) via Nemenyi’s procedure. It should be noted in all analyses *p* < 0.05 was used for significant differences. Five-point category scale data was reported as percentages and categorised into three groups: (i) bottom two boxes = very poor + poor; (ii) middle box = neutral; and (iii) good + very good.

## 3. Results

### 3.1. Initial Literature and Market Review on Protein-Fortified Products

The initial literature and market review identified food matrices often fortified with protein in relation to three key areas (literature, clinical and retail market) as outlined in Table 2. It is evident that a range of serving sizes (23–550 g and 100–500 mL) with differing protein content (0.9–50 g per 100 g or 100 mL) are regularly utilised; however, there is a lack of consistency in reporting, variability and/or a mix of protein sources (whey protein, milk and plant related or other), which can make product comparisons challenging. Moreover, common food matrices typically centred around familiar and popular products often in liquid or snack format.

### 3.2. Portion Size Questionnaire

**Figure 3:**
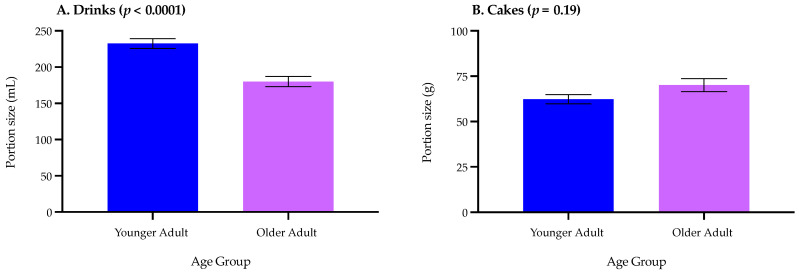
Consumers’ (*n* = 73) ideal portion size relating to (**A**) drinks and (**B**) cakes by age (younger adults *n* = 41 and older adults *n* = 32). Data reflected all drink and cake types presented (as defined in Table 1) and expressed as means ± standard error.

**Figure 4:**
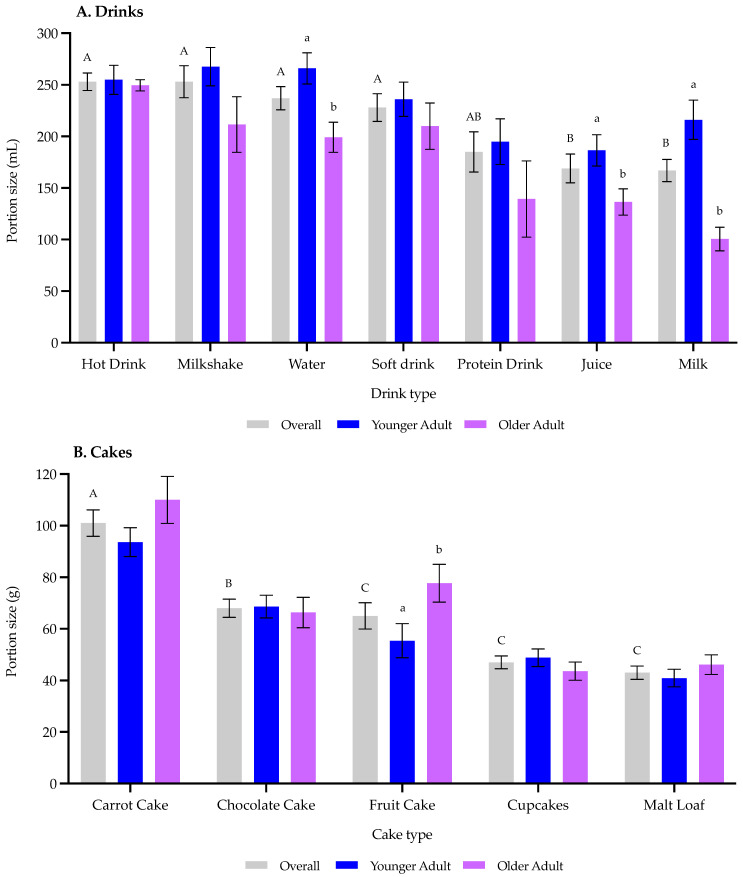
Consumers’ (*n* = 73) ideal portion size relating to (**A**) drinks and (**B**) cakes by type and age (younger adults *n* = 41 and older adults *n* = 32). Data expressed as means ± standard error. Differing letters reflect significance in preferred portion size as follows: (i) drink and cake types (capital letters) from Kruskal-Wallis and (ii) younger and older adults (small letters) via Mann-Whitney.

Older adults had a significantly lower (*p* < 0.0001) ideal portion size relating to drinks compared with younger adults (180 mL vs. 232 mL; Figure 3A). Overall, there were significant differences (*p* < 0.0001) between drink types where consumers reported a higher ideal portion size for hot drinks, milkshakes, water, and soft drinks (253–228 mL) than for protein drinks, milk, and juices (185–167 mL) (Figure 4).

There were no significant age-related differences (*p* = 0.19) for consumers’ ideal cake portion size (Figure 3B). Similar to drinks, there were significant differences (*p* < 0.0001) between cake types where consumers rated carrot cake (101 g) with an increased ideal portion size compared with other cake types (68–43 g) (Figure 4).

### 3.3. Protein Fortification Attitudes Questionnaire

**Figure 5:**
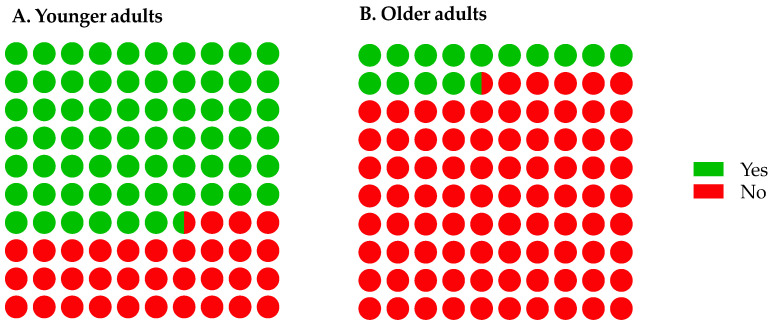
Overview of consumers’ (*n* = 67) consumption habits relating to protein-fortified products by age ((**A**) younger adults *n* = 39 and (**B**) older adults *n* = 28). “Yes” denoted consumers consumed protein fortified products: daily (4.5%), two-to-six times a week (4.5%), weekly (3.0%), one-to-three times a month (12%) and monthly (21%) and “No” highlighted never (55%) consumed protein-fortified products. Data expressed as % with each coloured circle demonstrates 1.0%.

**Figure 6:**
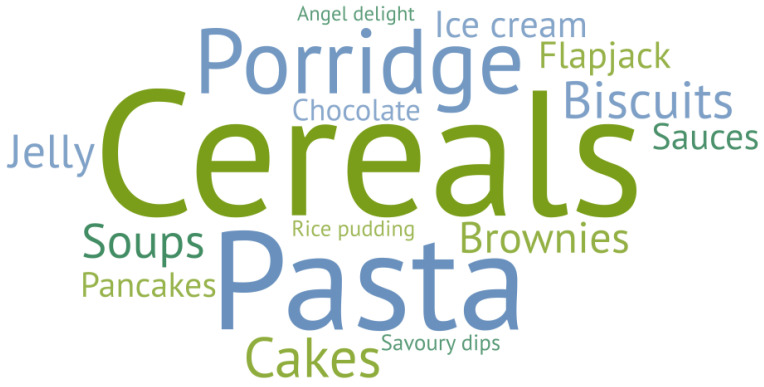
Word cloud outlining consumers (*n* = 67) most preferred products for protein fortification.

Older adults consumed protein-fortified products significantly less frequently (*p* < 0.0001) compared with younger adults (14% vs. 67%; Figure 5). There were significant differences (*p* < 0.0001) between consumers’ factors in terms of importance when selecting protein-fortified products were observed. Consumers ranked taste (1.7 a) and flavour (2.6 a) as key attributes followed by texture (3.8 b), cost (4.0 bc), smell (4.2 bc) and appearance (4.8 c) as the least important attributes (letters denote pairwise comparison from Nemenyi’s procedure). There were also significant differences (*p* < 0.0001) relating to preferences for protein fortification, where consumers selected cereals, pasta, porridge, cakes, and biscuits as the four most preferred product types compared with other product types (Figure 6).

**Figure 7:**
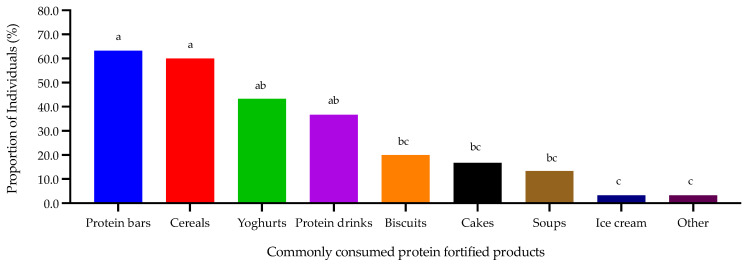
Consumers commonly consumed protein-fortified products (*n* = 30; consumers only answered this question if they regularly consume protein-fortified products). Data expressed as percentages with differing small letters reflecting significance from post hoc analysis.

**Figure 8:**
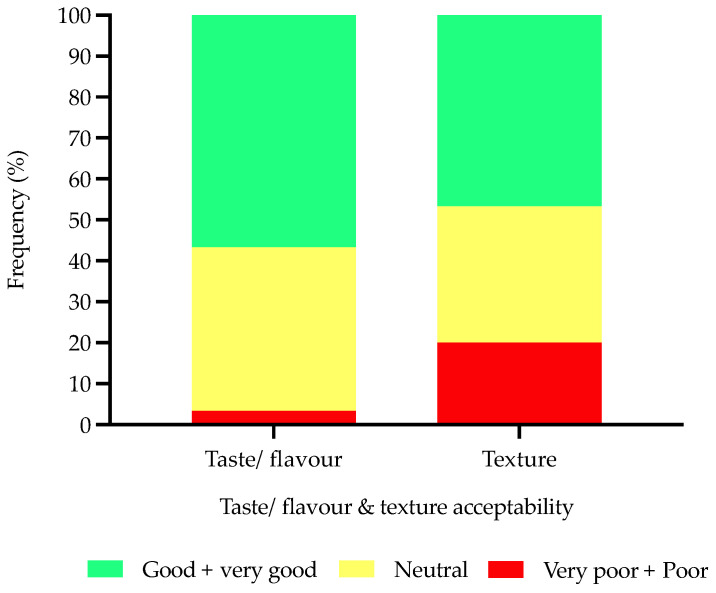
Consumers’ (*n* = 30) recalled perception ratings of protein-fortified products relating to taste/flavour and texture from a five-point category scale (data reported as percentages).

Consumers (*n* = 30) that regularly consumed protein-fortified products were asked a series of follow up questions. It was clear that these consumers typically consumed protein-fortified products at breakfast (53%) or as snacks in between meals (70%) rather than at lunch (20%) or as an evening meal (17%). Protein bars and cereals were significantly (*p* < 0.0001) the most consumed protein-fortified products compared with other product types (Figure 7). Consumers noted that the protein-fortified products they regularly consumed had either neutral (40%) or good taste/flavour (57%), whereas consumers were more critical towards the texture of these products (recalling more neutral (33%) or poor + very poor (20%) responses) (Figure 8).

## 4. Discussion

### 4.1. Initial Literature and Market Review on Protein-Fortified Products

The key themes from the initial product review related to familiar and popular products mainly in liquid or snack format with differences in serving size, protein content and protein sources; accordingly, making comparisons between products challenging. Moreover, the quality of the protein source is an important consideration for an ageing population and vital to maximising benefits. Therefore, corresponding serving sizes may need to vary between protein sources to get the same measurable output. Animal derived proteins (e.g., whey protein) have advantages (such as a complete essential amino acid profile, digestibility, and muscle protein synthesis) over plant derived proteins [9]. More specifically, whey proteins have been shown to trigger postprandial protein gain and muscle protein synthesis in an ageing population [8,11,55]. Overall, it is clear there is notable variability in terms of product types, protein sources (whey protein, milk and plant related or other) and protein quantity (0.9–50 g per 100 g or 100 mL) commonly utilised in protein-fortified matrices. Accordingly, next steps should focus on measurable and comparable outputs between different protein-fortified matrices with a variety of protein sources to ensure suitability for an ageing population. It is important to consider the nutritional and sensory aspects such as intake, muscle response and impact on appetite as well as product liking and ease of incorporation into the diet. More emphasis should be placed on catering for different individual preferences in order to deliver the relevant nutritional and health benefits. For example, if an older adult was to consume a plant derived protein biscuit, what should be the corresponding portion size compared with an animal derived protein biscuit? Moreover, it is apparent from Table 2 that products are typically more centred around animal derived protein sources than plant derived protein sources. However, with the increased demand to utilise plant derived protein sources; accordingly, it will be essential moving forwards to involve older adults in the design of such products. Additionally, understanding the impact of different protein sources on subsequent perception and acceptability as well as on nutritional and health outcomes. In addition, it could be suggested that future work should focus on developing an extensive database of suitable protein-fortified products for an ageing population considering different areas (e.g., literature, clinical, retail market, etc.), product types and protein sources. This information is key to ensuring a more tailored approach can be utilised, along with accurately estimating and/or calculating older adults’ needs as well providing product variety to promote consumption.

Older adults can have reduced appetite, modulated sensory sensitivity and oral impairments [29,30,31,32]; therefore, such changes mean products need to be developed and optimised accordingly. For example, (a) poor appetite could lead to reduced intake and unnecessary food waste, if the portion size is too large; (b) sensory sensitivity may change with age resulting in undesirable sensory attributes; and (c) oral impairments can make consuming products more difficult. In terms of key design implications from a sensory perspective, products should avoid being mouthdrying and adhesive (such attributes/properties may intensify with age), should be optimised for sweetness, fat, viscosity, and mouthfeel (e.g., soft texture, minimal dryness and easy to chew) and utilise familiar or popular flavours [29,56,57,58,59]. This should enable increased protein consumption (via familiar liquids, meals, or snacks) and a tailored and targeted approach could be key to promoting longevity and positive health outcomes in our ageing population.

### 4.2. Portion Size Questionnaire

There were age-related differences relating to consumers’ ideal drink portion sizes, where older adults preferred a smaller portion size compared with younger adults. It should be noted that standard ONS portion sizes are typically around 125 to 220 mL and the older adults in our study preferred drink portion size was 180 mL; therefore, this suggests there could be substantial wastage for products over this threshold. Accordingly, it is important that products are presented to older adults in a portion size that maximises protein consumption but also prevents wastage. Therefore, optimising volume to improve compliance is a key aspect in ensuring widespread benefits of such products [22].

In contrast to drinks and positively, there were no differences between age groups for the ideal cake portion sizes; interestingly, older adults did not select smaller cake portion sizes than younger adults. This suggests such familiar snacks may be suitable for protein fortification and subsequently help promote protein consumption in our ageing population. It is likely that older adults would be willing to consume up to around 70 g of cake, or more in some cases, depending on the cake type (i.e., carrot or fruit cake). As alluded to in Table 2, there is considerable variability relating to fortified snack portion sizes from 23 to 550 g. Specifically in cake products, example portion sizes were cited as 56–65 g in studies focusing on measuring protein intake in an ageing population [27,43,44] and 30–60 g in the retail market (predominately targeted at the sport, lifestyle, or health consumers). Therefore, this suggests there may be scope to increase protein-fortified cakes portion size to 70 g for older adults. More broadly, this supports previous work where familiar protein-fortified products can increase (i) protein intake and (ii) liking, in an ageing population [23,24,25,26,27,28,29]. For example, Beelen et al. noted consuming familiar protein enriched products (e.g., bread, cakes, soups, porridge, etc.,) can improve protein intake [27], whereas adding a popular topping (such as clotted cream) can increase consumer liking in whey protein-fortified scones [29].

Overall, this suggests snacks (such as cakes) may be beneficial in helping older adults consume more protein per serving size without adding excess volume. These products can easily be incorporated into the diets of older adults by providing a mid-morning or mid-afternoon snack and thereby enhance nutritional status. Future studies should consider measuring product leftovers from older adults so that portion size can be optimised and reflect older adults’ actual needs rather than self-reported data.

### 4.3. Protein Fortification Attitudes Questionnaire

Older adults in our study consumed protein-fortified products infrequently. It is likely older adults are often confused by, lack knowledge of, and/or are unaware of, protein requirements; therefore, this may explain why older adults consume these types of products less often [60]. Accordingly, this will contribute to lack of familiarity with such products, which can result in poor compliance and high wastage, especially with unfamiliar or disliked flavours [21]. Van Wymelbeke et al. demonstrated product type and familiarity can impact compliance where fortified brioche had increased compliance compared with ONS [52]. It should be noted that in some cases food neophobia could be a potential barrier to overcome for an ageing population, especially when consuming fortified products for the first time [61].

If consumers did consume protein-fortified products (mainly younger adults) these were typically consumed at breakfast or as snacks between meals; accordingly, this was matched by commonly consumed products (e.g., cereals and protein bars). However, there was scope for improvement for the taste/flavour and texture, which supports the literature that protein fortification can result in two key sensorial challenges, namely flavour and texture [40].

Consumers cited taste and flavour as key attributes for protein-fortified products and taste has been considered a driver for ONS dislike [62]. Therefore, product designers need to ensure that fortified products deliver on both taste and flavour to help promote protein consumption. A key finding from our study was that consumers considered cereals, pasta, porridge, biscuits, and cakes as products most preferred for protein fortification; such products can be regularly consumed at breakfast (cereals and porridge) or as snacks between meals (biscuits and cakes). These findings suggest that designing protein-fortified products based around the consumption moment (e.g., breakfast or snacks) could be a viable approach to encourage protein consumption for an ageing population. Moreover, this supports previous work, where it is likely older adults are more willing to consume protein-fortified products that are healthy, traditional, or familiar, coupled with key factors in product choice relating to natural, fresh and trust [60,63,64].

### 4.4. Practical Suggestions

It should be noted that the study sample size was relatively low; however, the practical implications of these findings can still help product designers and provide key directions for future protein fortification (such as breakfast and snacks). For example, older adults could consume a cereal fortified with protein for breakfast or a fortified snack between meals and thereby increase protein consumption. Additionally, it could be suggested that snacks be utilised to maximise protein intake since there were no age-related differences identified in the ideal portion size. Going forwards, a broader range of familiar protein-fortified products (such as bread, cereals, pasta, sauces, mashed potatoes, soups, chocolate, custard, rice pudding, ice cream and fruit juices) could be utilised. Testing a more extensive range of products could provide useful insights into older adults’ preferences and help identify further suitable age-related products for protein fortification. In addition, the older adults that partook in this study were healthy and community based; accordingly, investigating broader age ranges (such as over 75 years) and frailer populations would be useful to capture better portion sizes and/or preferences for an ageing population who are in need of increased protein intakes. Moreover, protein-fortified products are considered a well-tolerated and cost-effective approach, as well as falling within the food first remit [28,37]. Therefore, it is important that next steps include research utilising ecological validity to understand better older adults’ preferences and behaviour in practice (e.g., in community and clinical settings) to determine whether these products can be incorporated easily into everyday lives. It is fundamental that the target audience (i.e., older adults) is involved in the design process to tailor such products to encourage interest and maximise trust [60,63,64].

## 5. Conclusions

This paper combined an initial literature and market review to understand common protein-fortified food matrices with consumers’ views on portion size and protein fortification in order to help product developers optimise and design age suitable products. More specifically, fortified food matrices are typically centred around familiar and popular products in liquid or snack format; however, there is considerable variability in terms of product types, serving size and protein sources. Defining optimal portion sizes is an ongoing challenge; however, there were no age-related differences for the ideal cakes portion size, whereas there were for liquids. This implies cakes could be a better vehicle to promote protein consumption in an ageing population. A key priority is addressing the low intake of protein-fortified products amongst the ageing population. Emphasis should be placed on the consumption moment (such as breakfast or as snacks between meals) with preferences towards familiar cereals or snack-based products. Going forwards, research should focus on: (i) using older adults in the co-creation process; (ii) identifying the balance between portion size, wastage, and protein content; and (iii) developing products that can be readily incorporated into the everyday lives of older adults. More broadly, improved awareness and education, potentially in advance of reaching the ‘older adult’ stage, could encourage older adults to: (a) realise the benefits of protein consumption; and (b) incorporate such foods into their diet. This is especially relevant since older adults are typically unaware that they need more protein with age and should as a consequence result in more commercially fortified products being available in supermarkets.

## Figures and Tables

**Table 1:** Overview of portion size questionnaire by product type and portion size.

Products	Portion Size					
**Drinks**						
Water	25 mL	100 mL	175 mL	300 mL	375 mL	Comment
Juice	25 mL	100 mL	175 mL	300 mL	375 mL	Comment
Soft drink	25 mL	100 mL	175 mL	300 mL	375 mL	Comment
Milk	25 mL	100 mL	175 mL	300 mL	375 mL	Comment
Milkshake	25 mL	100 mL	175 mL	300 mL	375 mL	Comment
Protein beverage	25 mL	100 mL	175 mL	300 mL	375 mL	Comment
Hot drink	-	190 mL	220 mL	225 mL	260 mL	Comment
**Cakes**						
Carrot cake	-	-	50 g	100 g	200 g	Comment
Chocolate cake	-	40 g	70 g	138 g	-	Comment
Fruit cake	-	26 g	60 g	121 g	-	Comment
Malt loaf	-	30 g	61 g	91 g	-	Comment
Cupcakes	10 g	25 g	35 g	70 g	-	Comment

The portion sizes were based on the Carbs & Cals app (version 5.9.13, London, UK) and pilot testing within our laboratory.

**Table 2:** Examples of food matrices commonly fortified with protein within the literature, clinical settings and/or retail market.

	Food Matrix	Protein Source		Serving Size	Protein (g)	
					Serving Size	100 g or mL
**Literature-based studies**	Bread [26,27,41,42,43]	- ^†^		27–35 g ^†^	5.6–7.9	-
	Yoghurt [41,42]	WPC		100–250 mL	8.0–20.0	8.0 ^
	Fruit juices [26,27,43]	-		150–200 mL	10.0–10.6	-
	Soups [23,26,27,43]	-MP ^		75–79 g & 150 mL	6.9–10.1	-
	Mashed potato [26,27,43]	-		150 g	8.4–10.5	-
	Dairy drinks [27,43]	-		150 mL	10.1	-
	Cakes [27,43]	-		65 g	9.9	-
	Ice cream [27,43]	-		100 mL	10.0	10.0 ^
	Meat [27,43]	-		50–80 g	12.4–22.0	-
	Milkshake (orange) [44]	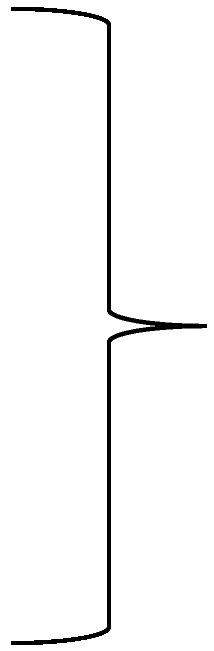	Combined approach of high protein products & protein powder (using whey, gelatin or pea protein)	150 g	16.0	-
	Chocolate cake [44]			56 g	7.6	-
	Pizza bun [44]			40 g	6.0	-
	Fruit salad [44]			65 g	8.4	-
	Bun [44]			40 g	4.9	-
	Cheese crackers [44]			10 g	1.4	-
	Sandwich (ham) [44]			40 g	5.3	-
	Jelly (apple & cream) [44]			50 g	9.5	-
	Enriched breakfast dishes [23]	MP		60–100 g	7.4–11.5	11.5 ^
	Enriched fish dishes [23]	MP		55–70 g	7.6–8.9	-
	Enriched meat dishes [23]	MP		55–75 g	6.5–7.9	-
	Enriched side dishes [23]	MP		47–75 g	6.1–7.7	-
	Enriched desserts [23]	MP		52–110 g	6.2–7.6	6.9 ^
	High energy and/or protein snacks [45]	-		-	0.0–6.0	-
	Protein rich ready meals [46]	-		500–550 g	30.5 (av)	-
	Protein rich dairy products [46]	-		30 g & 150 mL	5.1–11.6	-
	Biscuit [47]	WPI		40 g	5.0	12.4
	Sauces [48]	WPI		50 g	0.5–2.3	0.9–4.5
	Rye bread [49]	WPH & WPI		35 g	4.8–7.0	-
	Cream cheese [49]	WPH & WPI		25 g	2.9–3.0	11.6–11.9
	Muffins [50,51]	WP, AF & SF		100 g	9.1–14.1	9.1–14.1
	Brioche [52]	RP, MP		65 g	12.8	-
	Beverages [53,54]	WPC, WP		150–200 mL	20.7–24.0	-
**Clinical settings** **(ONS products)**	ONS *	MP, MPC, SP, MPI, SPI & CA		125–200 mL	11.2–20.0	5.6–10.0
	ONS soup *	MP, PP, SMP, MPC		150–200 mL	6.0–20.0	4.2–16.1
	ONS juice *	WP, WPI, MP, SPI		150–220 mL	7.8–11.0	3.9–16.2
	ONS yoghurt *	WP, MPI, SMP		125 g & 200 mL	9.3–15.0	5.9–7.5
	ONS dessert *	MP, MPC, MPI, SPI & CA		125 g	7.1–12.5	7.5–10.0
	ONS other *	WI & CH		118 mL	20.0	16.9
**Retail market-based products**	Protein milk *	WP, MP, MPC		330–500 mL	25.0	5.0–7.6
	Protein yoghurt *	Quark (milk)		150–200 g	15.0–22.0	10.0–11.4
	Protein cereals *	WPC, MPC, SMP & SPI		40–75 g	8.4–20.6	19.0–27.4
	Protein bars *	WPC, WPI, WP, SPI, MP, CA, MPI, WPH & PPI		30–65 g	4.5–20.0	15.0–34.0
	Protein flapjack *	WPC, WP, HWPI, SMP, MP, PP & HWHP		40–88 g	10.0–22.0	23.0–25.0 ^
	Protein balls *	WPI, WP, SP & MP		35–50 g	9.8–15.0	20.5–43.0
	Protein brownies *	WP, WPC, HWHP, CA, MP & SP		40–75 g	10.6–23.0	20.0–30.0
	Protein cakes *	WPC, WP, MPI, SPI & MP		30–60 g	7.9–15.0	25.0–26.0
	Protein pancakes *	WPC, SMP		45 g	16.0	35.5
	Protein cookies *	WPI, MP, MPI, SP & HWHP		59–75 g	13.0–38.0	18.0–50.0 ^
	Protein chocolate *	WPI		70 g	19.0–19.5	27.0–27.9
	Protein chocolate bars *	WPC, WP, SMP, MP & MPI		47–51 g	10.1–15.0	20.2–30.0
	Protein crisps *	WPI, WP, CA, MPI & SP		32–50 g	18.0–20.0	-
	Protein other *	WPI, WP, SMP, SPI & SPC		23–36 g	7.7–9.0	25.0–33.4
	Protein milk *	WP, MP, MPC		330–500 mL	25.0	5.0–7.6
	Protein yoghurt *	Quark (milk)		150–200 g	15.0–22.0	10.0–11.4

Dash (-) denotes not recorded within study. ^ represents reported by one study only or not reported by all studies within subset. Av outlines average and ONS represents oral nutritional supplement. Protein sources defined as follows: **whey protein** (WPC: whey protein concentrate; WPI: whey protein isolate; WPH: whey protein hydrolysate: WP: whey protein or powder; WI: whey isolate; HWPI: hydrolysed whey protein isolate); **milk related** (MP: milk protein; MPC: milk protein concentrate; MPI: milk protein isolate; SMP: skimmed milk powder; CA: caseinates derived); **plant related** (SP: soya protein; SPI: soya protein isolate or isolate soya protein; SPC: soya protein concentrate; SoF: soy flour; PP: pea protein; PPI: pea protein isolate; HWHP: hydrolysed wheat protein; AF: almond flour; RP: rice protein); and **other** (CH: collagen hydrolysate). ^†^ denotes small differences between studies: Beelen et al. noted protein source as soy and dairy based [26] whereas other studies the protein source was not reported [27,41,42]. Van Til et al. study only reported serving size as slice rather than in grams [41]. ONS and retail market products data * obtained from brands website. **ONS** describes six ONSs: 1 = Fortisip compact (Nutricia); 2 = Resource energy (Nestle); 3 = Fresubin protein energy drink; 4 = Altraplen compact (Nualtra); 5 = Ensure plus milkshake style; 6 = Aymes complete. **ONS soup** reflects six soups: 1 = Actasolve Savoury (Aymes); 2 = Vitasavoury (Vitaflo); 3 = Energis soup (Meritene^®^); 4 = Resource Soup (Nestle); 5 = Fortified soups (Apetito); 6 = Fresubin 2 kcal savoury. **ONS juice** notes six juices: 1 = Altrajuice (Nualtra); 2 = Ensure Plus Juice; 3 = Fresubin Jucy drink; 4 = Fortijuice (Nutricia); 5 = Aymes ActaJuce; 6 = Aymes ActSolve Smoothie. **ONS yoghurt** represents four yoghurts: 1 = Ensure plus yoghurt; 2 = Fortisip yoghurt; 3 = Fresubin YoDrink; 4 = Fresubin YOcreme. **ONS dessert** highlights six desserts: 1 = Forticreme Complete (Nutricia); 2 = Fresubin^®^ 2 kcal Crème; 3 = Ensure plus crème; 4 = Resource Dessert 2.0 (Nestle); 5 = Nutricrem (Nualtra); 6 = Aymes Actacal crème. **ONS other** signifies jelly (Prosource Jelly; Nutrinovo). **Protein milk** denotes four milks: 1 = Protein chocolate milk (Arla); 2 = Protein chocolate milk (Dale Farm); 3 = Protein chocolate milk (Maximuscle); 4 = High protein chocolate (For Goodness Shakes). **Protein yoghurt** reflects three yoghurts: 1 = protein yoghurt (Arla); 2 = Lindahl Kvarg (Nestle); 3 = Protein 22 (Graham’s). **Protein cereals** notes four cereals: 1 = Protein granola (MyProtein); 2 = Protein granola (Lizi’s); 3 = Protein oats (oomf); 4 = Protein porridge (Fuel). **Protein bars** represents four bars: 1 = Cereal bar (MyProtein); 2 = Granola bar (MyProtein); 3 = Carb killa (Grenade); 4 = Diet whey bar (PhD). **Protein flapjack** highlights four flapjacks: 1 = Snickers protein flapjack (Mars); 2 = Oats & whey protein flapjack (MyProtein); 3 = Protein flapjack (Oatein); 4 = Protein flapjack (Bulk). **Protein balls** signifies four balls: 1 = Choc protein balls (MyProtein); 2 = Energy bites (MyProtein); 3 = Protein ball (The Protein Ball Company); 4 = Chocolate balls (Bulk). **Protein brownies** denotes four brownies: 1 = Protein brownie (MyProtein); 2 = Protein brownie (Mountain Joe’s); 3 = Protein brownie (The Protein Works); 4 = Oatein brownie. **Protein cakes** indicates two cakes: 1 = Pop Roll (MyProtein); 2 = Protein cake (PhD Smart). **Protein pancakes** expresses protein pancake (Nano ä). **Protein cookies** highlights four cookies: 1 = Protein cookie (MyProtein); 2 = Baked cookie (MyProtein); 3 = Protein cookie (Quest); 4 = Protein cookie (Oatein). **Protein chocolate** reflects two chocolates: 1 = Protein chocolate (MyProtein); 2 = Protein chocolate (Cocoa+). **Protein chocolate bars** describes three chocolate bars: 1 = Mars hi-protein chocolate bar; 2 = M&M’s hi-protein bar; 3 = Snickers protein bar. **Protein crisps** notes two crisps: 1 = Protein crisps (Quest); 2 = Protein crisps (GOT7). **Protein other** represents two crispies: 1 = Protein choc crispies (MyProtein); 2 = Protein crispies (The Skinny Food Company).

## Data Availability

The data presented in this study is available on request from the corresponding author.
